# Snake-bites

**Published:** 1843-01

**Authors:** 


### SNAKE-BITES.

A notice sometime since, that we were anxious to obtain information concerning the bites of our venomous snakes, has brought us several communications; some of which enclosed specimens of plants, said to be efficient remedies. We thank their authors, and solicit a continuation of their favors; but take this opportunity to say, that our object is rather to investigate the pathology of snake-bites, than the remedies on which reliance, in different parts of the country, is placed for their cure. In reference to the former, several curious questions present themselves, to which we ask attention:

1. Are the bites of all our venomous snakes, from Michigan to Florida, equally pernicious?

2. Do they all generate the same symptoms, differing only in degree? Or are there characteristic peculiarities in the diagnosis of each?

3. Are any temperaments or idiosyncrasies a protection against the poison? What is the proportional number of deaths?

4. Does the wound ever inflame and suppurate?

5. Does the bitten limb swell in a few minutes, or within an hour or two, after the bite is inflicted?

6. When ecchymoses appear, what is the earliest period after the bite, and are these spots as common in the skin generally, as they are on the wounded limb?

7. From which of the mucous membranes is hæmorrhage most frequent, and in what proportional number of cases does it occur? And what is the character of the blood?

8. When death ensues, what are the *post-mortem* appearances, external and internal; and in what state is the blood, as to its colour and relative proximate elements? Does the body undergo decomposition earlier than usual?

9. Is there an annually recurring irritation in the bitten part, or in the constitution?

10. What domestic animals are proof against the poison?

11. Does the "Blowing Snake" or Hissing Adder (*Heterodon Platirhinos* of Holbrook) emit a poisonous vapour?

12. What is the most effectual treatment, for the bites of all our snakes? What is the modus operandi of ammonia? Does it act as an antidote to the poison, or by its stimulating properties remove the constitutional irritation?

The attention of our brethren, in regions where poisonous snakes abound, is respectfully invited to these inquiries. D.

